# Participating in a new group and the identification processes: The quest for a positive social identity

**DOI:** 10.1111/bjso.12340

**Published:** 2019-10-11

**Authors:** Diana Cárdenas, Roxane de la Sablonnière

**Affiliations:** ^1^ Universiteit Utrecht Utrecht The Netherlands; ^2^ Université de Montréal Québec Québec Canada

**Keywords:** additive and subtractive identification patterns, identification, immigration, participation, positive social identity, social identity theory

## Abstract

Immigrants experience identity shifts; they can identify with the new cultural group and, sometimes, identify less with their group of origin. Previous research suggests that participation in the new cultural group predicts these two identity shifts. However, these studies have exclusively used correlational methodologies. Furthermore, previous research ignored that when a group is negatively valued, individuals may not identify with it, even after participating in it, to preserve a positive social identity. This article tests with an experimental methodology whether participation recreated the identity shifts previously identified (greater identification with the new group and lower identification with the group of origin when perceiving dissimilarity). Furthermore, it tested how a group's value impacted these identity shifts following participation. Immigrants in Quebec (*N* = 184) either participated in Quebec's culture (watched hockey) or did not (watched basketball). Quebec's value was manipulated by changing whether Quebec won, tied, or lost the game. Compared to watching basketball, watching Quebec's team win or tie showed the hypothesized identity shifts, illustrating the importance of the new group's value when participating.

Roughly 20% of the Canadian population is born in another country (Morency, Caron Malenfant, & MacIsaac, 2017). High immigration rates are also seen in the United States (13%; Grieco *et al*., 2012), Australia (28.2%; Australian Bureau of Statistics, 2017) and the United Kingdom (14%; White, 2017), showing how questions on immigration matter across borders. These newcomers often develop a new repertoire of behaviours typically associated with the new cultural group, as they can now participate in this new group (or engage in behaviours or actions typically observed in the new cultural group; Cárdenas & de la Sablonnière, 2017). They also find that their cultural identities are subject to shifts, as they can identify with a new cultural group, but also, sometimes, identify less with their group of origin.

Recent research suggests that both participation in the new group and identity shifts are related. In four studies employing surveys and questionnaires, participating in the new cultural group predicted higher levels of identification with it (Cárdenas *et al*., 2018; see also Cárdenas & de la Sablonnière, 2017); higher identification with the new group in turn predicted lower levels of identification with the group of origin when individuals perceived dissimilarities between these groups (Cárdenas *et al*., 2018). The predictive ability of participation on identification shifts is thus far observed with correlational methodologies.

However, without experimentally manipulating participation in the new group and observing its effects on identity, it remains unknown whether participation and identification shifts simply co‐occur or whether participation has the potential to impact identification. Only controlled experimental designs can isolate participation in the new group and determine its causal role on identity shifts.

Not only does previous research prevent us from assuming causality, but also it neglected to consider that individuals wish to belong to groups with a positive value (Tajfel & Turner, 1979). Indeed, the value attributed to a group impacts individuals’ motivation to be associated with the group. For example, individuals belonging to groups with negative characteristics tend to disassociate from them (Jackson, Sullivan, Harnish, & Hodge, 1996; Sachdev & Bourhis, 1987). Thus, the positive impact of participation on identification with the new group might be conditional to the new group's value; an immigrant may not be inclined to identify with a negatively valued new group, even after participating in it.

The present research has two goals. The first goal was to use an experimental design to test whether participating in a new group (as opposed to not participating) triggers identity shifts (i.e., increased identification with the new group which will decrease identification with the group of origin when little similarities between groups are perceived). The second goal was to test whether the value of the new group determines the impact of participation on identity changes. As such, the current research offers insight not only into how behaviours directly impact the identity of immigrants, but also into how individuals seek to fulfil their desire to belong to positively valued groups in the context of immigration.

## Participating in the new group, identification with the new group, and identification with the group of origin

Previous studies have focused on how personal (personality traits; Redfield, Linton, & Herskovits, 1936; sharing goals with the new group; Zhang & Chiu, 2012), cognitive (need for cognition; Kashima & Pillai, 2011), and contextual/environmental factors (e.g., discrimination; de Vroome, Verkuyten, & Martinovic, 2014) can help or hinder immigrants’ identification with a new group (for more factors, see Berry, 2001). However, these factors are frequently outside the control of the individual, and specific social environments, personality traits, and cognitions can be difficult to change. Cárdenas and de la Sablonnière (2017) proposed a factor more accessible and modifiable by the migrant population itself: participating in the new cultural group (e.g., engaging in cultural traditions, social/work/education activities, and relationships with members of this new group; Cárdenas & de la Sablonnière, 2017). It was proposed that participation in a new group activates two psychological mechanisms (Cárdenas & de la Sablonnière, 2018): the perception that one is a prototypical member of this group (Cárdenas & Verkuyten, 2019; Hogg, 2005; Turner, 1987) and the need for consistency between one's actions and identity (Cialdini, 2009; Fiske & Taylor, 2013; see also Swann, 1983).

The relation between identification and participation was initially tested among Latin American immigrants to Canada (Cárdenas & de la Sablonnière, 2017). Three path analysis models were compared: The first tested whether participation in the new group and identification with it were correlated (but did not predict each other); the second tested whether identification predicted participation; and the third model tested whether participation promoted identification. This last model, where participation in the new group predicts higher identification with it, received the strongest support, a finding replicated with a qualitative methodology (Cárdenas & de la Sablonnière). A second series of studies conducted in the context of globalization not only replicated these findings, but they also showed that participation in a new group could have a trickledown effect on the identity of origin via the newly acquired identity (Cárdenas *et al*., 2018). Specifically, the current position in acculturation literature, the branch of psychology examining how individuals change following cultural exchanges, is that adding a new identity does not have negative consequences for the identity of origin (i.e., that the relation between these identities is positive or non‐existent; Berry, 1997). This has been termed the additive identification pattern (de la Sablonnière, Amiot, Cárdenas, Sadykova, Gorborukova & Huberdeau, 2016). However, correlational studies also show evidence for a subtractive pattern, in which increased identification with a new cultural group is related to lower identification with the group of origin (de la Sablonnière *et al*., 2016; Repke & Benet‐Martinez, 2017; see also Fleischmann & Phalet, 2018). This subtractive pattern is predicted by perceived rejection from the groups (e.g., perceived discrimination, Fleischmann & Phalet, 2018) and, importantly, perceived dissimilarities between groups and their characteristics. More precisely, being a member of two cultural groups with few perceived similarities can make it difficult to prone and endorse both groups simultaneously and to the same extent (Cialdini, 2009). At the identity level, this should be reflected in a subtractive identification pattern. Indeed, previous research has found that perceived group differences (Cárdenas *et al*., 2018) and in specific group attributes (e.g., status; de la Sablonnière *et al*., 2016; linguistic characteristics, Study 4 in Cárdenas *et al*., 2018) predict the subtractive pattern. In the context of participation, the results from four correlational studies found that participating in the new group predicted greater identification with the new group, which in turn negatively predicted identification with the group of origin when dissimilarities between the groups were perceived.

Overall, these findings highlight how engaging in behaviours typical of the new group predicts the way individuals relate to their new groups and, consequently, to their groups of origin. However, previous methodologies (correlational or qualitative designs) prevent us from assuming that participation causes identity shifts. Thus, it is impossible to establish whether participation in a new group increases identification with the new group, an increase that is associated with the additive/subtractive patterns of identification.

Correlational studies, and particularly cross‐sectional methodologies, are the preferred methodology in acculturation psychology (Ryder & Dere, 2006). This methodology, unlike experiments, allows researchers to examine culture in its natural setting. Experiments, on the hand, require bringing culture and the changes it produces into the laboratory, an important challenge considering the abstract nature of culture. Nevertheless, it is possible to study cultural changes in the laboratory. For example, the independent or interdependent self‐construals and its cross‐cultural differences have been experimentally manipulated by asking individuals of Chinese or North American origin to think about either the commonalities or the differences with their friends and families (Trafimow, Triandis, & Goto, 1991). In another line of research, the clarity of cultural identity was manipulated using computer‐mediated communication (Usborne & Taylor, 2012). While these experiments might not replicate all the elements of culture, the components they do manipulate further the field's understanding of how culture impacts individuals by isolating one specific factor and testing its causal impact on individuals. Considering, first, the correlational evidence that participation predicts identification shifts, and, second, that the impact of culture can be examined with experimental designs, the first goal of this article was to use an experimental methodology to ascertain whether participation in the new group can increase identification with the new group, leading to the additive/subtractive identification patterns.

## The need for a positive social identity and participation in the new group

Not only is previous research limited to predictive instead of causal links, this literature has also assumed that the moment an individual participates in the new group, the psychological mechanisms that promote identification with the new group will be activated, triggering the identity changes previously observed (the subtractive pattern if differences are perceived). In other words, it assumes that the effect of participation on the new cultural identity and hence the cultural identity of origin are unconditional. This assumption is unwarranted given individuals’ need for a positive social identity.

According to social identity theory (Tajfel, 1981, 1982; Tajfel & Turner, 1979), individuals are inherently motivated to have a positive social identity. To do so, they seek membership in groups that have a positive value. Indeed, the ‘value‐laden nature of group membership’ is an essential aspect of group membership (Taylor & Moghaddam, 1994, p. 78), guiding the perceptions and actions of the individuals. When individuals’ need for a positive social identity is not satisfied by the group, they will negotiate their membership in this and/or other social groups to enhance their social identity. This is done by identifying less with their current group, leaving it, and/or joining a group with positive characteristics (Mummendey, Kessler, Klink, & Mielke, 1999; Sachdev & Bourhis, 1987; Tajfel, 1981; Tajfel & Turner, 1979). By putting either psychological (recategorizing themselves) or physical (social mobility, physically leaving their group) distance between the negatively evaluated groups and themselves, their social identity is protected (e.g., Bettencourt, Dorr, Charlton, & Hume, 2001; Jackson *et al*., 1996; Mummendey *et al*., 1999; Sachdev & Bourhis, 1987). For example, in a study by Sachdev and Bourhis (1987), participants were explained that creativity was an important asset for their academic and professional life; they were then randomly assigned into one of two conditions after completing a fake creativity test: a high‐value/low‐value condition or a neutral condition. In the high‐value/low‐value condition, participants were randomly assigned (without their knowledge) into either a high‐creativity group or a low‐creativity group. In the neutral condition, participants were divided into two average and equally creative groups. After this random assignment into conditions and creativity groups, participants’ identification with their group was assessed. Participants in the high‐creativity group identified more with their group than participants in the medium‐creativity group and than participants in the low‐creativity group. When asked how much they believed participants in the high‐creativity group and low‐creativity group identified with their respective groups, participants expected those in the high‐creativity group to identify more with their group and those in the low‐creativity group to identify less with it. Overall, research from a social identity theory framework has supported the contention that individuals will identify less with negatively valued groups.

The value attributed to groups (and thus its contribution to a positive social identity) has also received some attention in immigration/acculturation literature. For example, Bourhis, Moïse, Perreault, and Senécal (1997) presented a model explaining how immigrants adopt a new culture and how they continue to enact their culture of origin. The central question of this model is whether immigrants consider that it is valuable to adopt the culture of the new country. If it is not considered valuable, then immigrants will engage in strategies that reject the new culture.

To summarize, there is evidence that individuals will identify less with groups that have a negative value. In contexts in which individuals participate in a new cultural group, the negative value associated with the group might cancel the positive impact of participation on identification. In other words, an immigrant living in Canada and participating in the Canadian group should normally identify more with Canadians. However, if this immigrant perceives that the Canadian group has a negative value, then the effect of participating in Canadian culture may be cancelled by the negative value. In this case, participation would not help individuals’ self‐perception as prototypical Canadians, hence not increasing identification with Canadians.

## Context of the study

By using an experimental design, the present research will test whether participating in the new group increases identification with the new group and whether this increase in identification with the new group would result in an additive (i.e., non‐negative relation) versus a subtractive (i.e., a negative relation) pattern of identification depending on perceived dissimilarities in the groups’ characteristics (i.e., a moderated mediation). Furthermore, we extend previous findings by testing whether the value attributed to the group in which one participates can determine whether participation triggers the identification changes previously described.

To test these hypotheses, participation is operationalized as sport viewing. The power of sports to move people has been long acknowledged. In Roman times, ‘bread and games’ is said to be all that was required to keep the populace happy (Juvenal as cited in Mastin, 2009). Today, the Olympics hold the eyes of the entire world for 2 weeks (Roxborough, 2016), soccer/football games draw blood from spectators (e.g., ‘Hincha del América muere tras riña entre barras en Cali’, 2017), and losing hockey's Stanley cup results in a city riot (Riots erupts in Vancouver after Canucks loss, 2011). Clearly, sports hold a power over communities and cultural groups, to the extent that they have become an important expression of cultural (Bernache‐Assollant, Chantal, Bouchet, & Lacassagne, 2016) and national identities (Maguire & Tuck, 2005). This holds even for spectators, as observing a game has ‘the element of ritual and emotional appeal capable of sustaining the “imagined community” of the nation’ (Houlihan, 1997, p. 121).

Such is the case of hockey in the province of Quebec, Canada. In this Canadian province, four out of ten Quebecers consider themselves fans of the Canadians of Montreal, a professional hockey team playing in the National Hockey League (or NHL; Côté, 2012). In 2016, roughly 1.4 million televisions sets in Quebec (with a total population of 8.18 million) tuned in to the first game of the Canadians in the NHL Series (Lemieux, 2015). In an in‐depth demographic analysis of Quebec, Côté (2012) argued that hockey and the Montreal Canadians took the place of the Catholic religion in defining Quebec's identity, putting hockey at the very centre of Quebec's society. This becomes manifests in the almost religious observance of watching hockey games, a typicality easily observed by outsiders (e.g., Ransom, 2014).

In the present study, we make use of watching hockey as a typical Quebecer behaviour to experimentally manipulate participation in the new culture. More specifically, watching a 5‐min long video of the Montreal Canadians playing against the Rangers of New York was conceptualized as participating in Quebecer culture. In contrast to hockey, watching a video of a basketball game where the Miami Heat played again the Dallas Mavericks was operationalized as not participating in the Quebecer culture. Basketball does not enjoy the same popularity as hockey in Quebec (particularly when the study was conducted in 2016), as no city in Quebec has a basketball team. Professional basketball is also rarely watched in Quebec (even though this changed in 2019; The story behind the Toronto Raptors 'We The North' campaign, 2019). Considering how basketball parallels hockey (e.g., team sport, one item is passed from teammate to teammate, and scoring goals/hoops is the aim) yet lacks prominence in Quebec's culture and media, it was chosen as a control to participation.

The specific hypothesis tested is that immigrants watching hockey (as opposed to watching basketball) will identify more with Quebecers and consequently identify less with their country of origin if they perceive little similarity between hockey and their national sport (replicating the moderated mediation from Cárdenas *et al*., 2018). However, this moderated mediation should only occur when the Montreal Canadians are winning (i.e., positive value of Quebec) and tying (i.e., neutral value of Quebec) their game. When the Montreal Canadians lose the game (i.e., negative value of Quebec), we would not expect identification with Quebecers to increase, as the positive effect of participation is counteracted by the negative effect of having a negative value (i.e., losing). Thus, the moderated mediation to predict identification with the country of origin should also be inexistent.

## Method

### Participants

A total of 199 first‐generation immigrants to Quebec, Canada, were recruited to participate in this study after ethics approval was obtained from the university's ethics board to ensure ethical behaviour. Participants were recruited via a snowball sample and by posting messages at a university campus, at community centres, and via Facebook groups. Of this initial sample, one participant was removed because the person was born in Canada, another participant was removed because the individual had Quebecer parents and lived in the United States for only 2 years, and thirteen were removed because they guessed the goal of the study. A final sample of 184 was kept for analysis.1The data set is available at https://zenodo.org/record/3379831. Because of the sampling strategy employed in this study, most participants were students (62.5%). Thus, the average age of current participants (*M *=* *29.60, *SD *=* *12.20) was lower than the age of recent migrants (*M* recent migrant population = 31.7) and the total migrant population in Canada (*M* total migrant population = 47.4; Statistics Canada, 2018). In addition, most participants were women (63.1% were women). These proportions reflect the mix between student (young and mostly women) and non‐student population in our sample. Participants were from 62 countries of origin, ranging from Algeria to Vietnam; the country of origin most often reported was France (20.1%). In terms of the United Nations geoscheme of world regions and subregions, most participants came from Western Europe (26.5%; Eastern Europe, 12.9%; North Africa, 12.4%; South America, 8.1%; Western Asia, 7.5%; Caribbean, 7%; Central America, 6.4%; Central Asia, 6.4%; Southern Asia, 3.2%; Eastern Asia, 2.6%; South‐eastern Asia, 2.6%; Middle Africa, 2.1%; Western Africa, 2.1%; North America, 1.1%; Eastern Africa, 1%; Southern Europe, 1%; Northern Europe, 0.5%; Melanesia, 0.5%).

Most participants had become Canadian citizens (*n *=* *118). The mother tongue most often reported was French (*n *=* *57), followed by Arab (*n *=* *33) and by Spanish (*n *=* *27). On average, participants had resided in Canada for 146.81 months (*SD *=* *124.93). Given the average length of stay, the migrants in our sample are not experiencing in our study their first opportunities to participate in Quebec culture. However, research suggests that for first‐generation migrants the receiving country continues to be the ‘new’ group. For example, identification with receiving country is lower in first‐generation migrants than in later generations (e.g., Giuliani, Tagliabue, & Regalia, 2018; Ono, 2002; Rumbault, 2010) and identification with the country of origin is generally higher than identification with the receiving country (e.g., Van Heelsum & Koomen, 2015). Together, the identification differences (between generations; between new country versus country of origin) highlight the possibility for further and continued growth in identification with receiving country for first‐generation migrants. Thus, this sample of first‐generation immigrants allows us to test our hypotheses among migrants with previous opportunities to participate.

### Procedure

The study was presented to participants as an investigation concerning the impact of immigration and sports on the self‐concept and well‐being. The online survey, hosted by Fluid Survey, began with a consent form, followed by questions concerning participants’ involvement in hockey, basketball, and golf (this last sport was added to avoid raising suspicion about the specific goal of the study). The questions asked to what extent individuals were fans of hockey and how often they played hockey, watched hockey, and watched hockey with Quebecers. These same questions were then asked about the two other sports. Participants were then randomly assigned to one of four conditions in which a video of approximately 5 min was presented. The video summarized either a hockey or a basketball game, presenting the main goals/points of the games, along with important defensive or offensive plays that did not result in goals/points. Participants in the hockey loss condition (*n *=* *47) watched the summary of a game in which the Montreal Canadians lost to the New York Rangers by a point; in the hockey win condition (*n *=* *46), the Montreal Canadians wan against the Rangers by a point; to create the hockey tie condition (*n *=* *51), and since NHL games do not end in ties, the same video as in the hockey win condition was set to end while the Montreal Canadians and the Rangers were tied. Lastly, in the basketball condition (*n *=* *40), participants watched a summary of a basketball game between the Miami Heat and the Dallas Mavericks. To ensure an appropriate comparison with the hockey tie condition, the basketball video ended when the two teams were tied. This also ensured that no positive or negative value influenced the results of this condition. For the four conditions, summaries were preferred over 5‐min periods of the games because they allow participants to experience the games in a condensed way; summaries also allow for multiple scores in hockey games (which may not necessarily occur in any given 5‐min period). We ensured that participants watched the whole video by allowing them to move to the next part of the survey only after the 5‐min video had elapsed. Participants then answered measures of identification and of similarity.

The two main conditions, the basketball (control) condition and the hockey win condition, were pretested before the data collection by having immigrants either watch the basketball video (*n *=* *8) or the hockey win condition (*n *=* *14) in the laboratory, followed by measures of identification with Quebecers (ranging from 1 = Totally disagree to 5 = Totally agree). Results showed that the hockey win condition had a higher mean of identification with Quebecers (*M *=* *2.07; *SD *=* *1.14) than the basketball condition (*M *=* *1.50; *SD *=* *0.76), a difference that was not significant given the small sample, *t*(20) = 1.26, *p *=* *.222. These results were then utilized to estimate the largest effect size we should expect (Cohen's *d *=* *.59). After this, G‐power was used to determine the sample size for the main study (*N *>* *178), given a medium observed effect size, the four conditions, and six control variables (status, legitimacy, their interaction, playing basketball, playing hockey, and contact with Quebecers). To ensure that this minimal number of participants was achieved for the results, we aimed to recruit 200 participants.

### Measures

Online studies that are short (around 9 min) have lower participant drop‐off and less random answers (Qualtrics, 2019). Considering how 5 min were being taken by the video, we opted to use single‐item measures in most of the questionnaire to maintain a short‐length study.

#### Identification

The present research took place in Quebec, Canada. The province of Quebec is different from other Canadian provinces in two ways. First, it has a distinct identity from the Canadian identity, greatly based on the French heritage and language. Second, Quebec shares jurisdiction with Canada in terms of the immigration: Quebec selects the immigrants it desires, and Canada officially accepts them in the country (Gouvernement du Québec, 2006). The fact that Quebec has distinct and unique identity compared to Canadians, and that it is in control of its immigration, has as a consequence that immigrants in Quebec quickly distinguish the Canadian from the Quebecer group, recognizing that the primary identity of their environment is the Quebecer identity. For this reason, in the present research, we focused on identification with Quebecers.

The Single Item Identification Scale (Postmes, Haslam, & Jans, 2013; Reysen, Katzarska‐Miller, Nesbit, & Pierce, 2013) was used to measure identification with Quebecers and with country of origin. This scale was chosen because it taps at self‐categorization and should be sensitive to our manipulation. Participants answered the following question using a scale ranging from 1 (*Strongly disagree*) to 7 (*Strongly agree*): In general, I identify with [Quebecers/members of my country of origin].

#### Similarity

Based on Cárdenas and de la Sablonnière (2017), similarity between cultural practices was measured with a single item stating that Hockey was similar to the sport of their country of origin (1 = *Strongly disagree* to 7 = *Strongly agree*).

In addition to these main variables, seven control variables were utilized in this study to ensure that the results observed were not due to differences in these variables. The control variables are playing basketball, playing hockey, time since immigration, contact with Quebecers, status, legitimacy, and the interaction between status and legitimacy.

#### Playing Basketball and Hockey

To measure how often participants played hockey and basketball, they were asked how often per month they played hockey (1 = *Never* to 6 = *10 times per month or more*) and basketball (same Likert scale).

#### Time since immigration

To assess how much time participants had spent in Canada, they were asked to report the number of months since they had immigrated to Canada.

#### Status and legitimacy

Status and legitimacy predict the subtractive identification pattern (de la Sablonnière *et al*., 2016). To ensure that similarity of characteristics predicts the subtractive pattern beyond these variables, status, legitimacy, and their interaction were controlled for. Status was evaluated by asking participants to evaluate the status of Quebecers compared with the people of their country of origin (1 = *Quebec's status is very weak* to 7 = *Quebec's status is very strong*). Following this question, participants were asked to extent to which this situation was legitimate (1 = *Not at all legitimate* to 7 = *Very legitimate*).

#### Contact with quebecers

Contact with a new group predicts greater identification with the new group (e.g., Gartner *et al*., 2000; Munniskam, Verkuyten, Flache, Stark, & Veenstra, 2015). To ensure that contact did not affect our results, contact with Quebecers was controlled for. It was evaluated by asking participants how many of their friends, their colleagues, and their neighbours were Quebecers, as well as how much contact they have with Quebecers in general (1 = *None* to 4 = *A lot*; alpha Cronbach = .76).

### Analysis plan

First, we ensured that the data met the necessary assumptions for the analyses (preliminary analysis). The main analyses are conducted in the second, third, and fourth steps, which overall test the hypothesized moderated mediation using the equations developed for PROCESS (Hayes, 2013) in path analysis (Stride, Gardner, Catley, & Thomas, 2015) Specifically, in the second step, we examined whether the overall model fits well the data (based on the fit indices; chi‐square *p *>* *.05 RMSEA < .05; and CFI > .95). Only if the model was acceptable could we examine the specific path (or relation) between variables and indices of moderated mediation, which altogether test our hypotheses?

In the third step, the specific paths within the model were examined. Specifically, to test the effects of participating in a group that is positively, neutrally, or negatively valued (i.e., watching hockey win, tie, and lose) over not participating (i.e., watching basketball), three dummy variables were created. These variables compare each of the hockey conditions to the basketball (control) condition. The three dummy variables were set to predict identification with Quebecers (*a*_1_ = Basketball/Hockey lose; *a*_2_ = Basketball/Hockey tie; *a*_3_ = Basketball/Hockey win). In turn, identification with Quebecers was set to predict identification with the country of origin (*b*_1_). These paths test the mediating effect of the participation conditions (versus no participation) on identification with country of origin via identification with Quebecers. To test whether similarity moderated this relation, similarity (*b*_2_) and the interaction between similarity and identification with Quebecers (*b*_3_) were also set to predict identification with the country of origin. Status, legitimacy, their interaction, and the number of times per week that participants played Basketball and Hockey were added as control variables (predicting identification with country of origin and covarying with identification with Quebecers). In the fourth and last step, three indices of moderated mediation (one per dummy variable) were calculated to test whether the mediating effect of participation (versus no participation) on identification with country of origin via identification with Quebecers was moderated by similarity.

Lastly, in the fifth step, a set of additional analyses are included. These test whether a reversed order of effects was supported by the data (manipulation decreases identification with country of origin and in turn identification with Quebecers) and whether participation in the new cultural group by watching hockey could be empirically distinguished from identity salience.

## Results

### Preliminary analysis

Data were inspected for missing data, univariate and multivariate outliers, and data normality. No participant had missing data in the main variables of the study, and no univariate and multivariate outliers were identified. The main variables had normal ranges of skewness and kurtosis (Kline, 1998); however, closer inspection of the similarity variable revealed a U distribution of the scores, with 26.6% of participants selecting the lowest value (1 in the Likert scale) and 24% selecting the highest value (7 in the Likert scale). The remaining 50% of the sample was distributed similarity between the middle values (2–6 in the Likert scale). Considering the lack of normality in this variable, MLR analysis was employed in MPLUS. This option permits the usage of non‐normal variables by using maximum‐likelihood estimates with robust standard errors (Wang & Wang, 2012).

Lastly, two participants identified hockey as the sport of the country of origin. One participant who watched the basketball video also identified basketball as the national sport of their country. Considering that watching a video of their national sport could have potentially impacted our results, the analysis was conducted with and then without these three participants; the results remained the same when removing the three participants, and hence, they were kept in the following results. For means, standard deviations, and correlations, see Tables 1 and 2.

**Table 1:** Means and correlations

	Mean (*SD*)	1	2	3	4	5	6	7	8
1. Identification with Quebecers	4.51 (1.69)	–	−.39***	.07	−.07	.10	.04	.01	.35***
2. Identification with country of origin	5.38 (1.56)		–	.06	−.06	−.16*	.04	.14†	−.09
3. Similarity	3.91 (2.33)			–	−.05	.05	.02	.15*	.07
4. Status	4.56 (1.01)				–	.20**	.04	−.04	−.16*
5. Legitimacy	4.33 (1.49)					–	.04	−.19**	−.07
6. Play Hockey	1.15 (0.53)						–	.10	−.02
7. Play Basketball	1.33 (0.67)							–	−.10
8. Months since immigration	146.81 (124.59)								–

Note

^†^ *p *<* *.10;*p <* *.05; **p <* *.01; ***p <* *.001.

**Table 2:** Means across conditions

Conditions	*n*	Identification with quebecers mean (*SD*)	Identification with country of origin mean (*SD*)
1. Basketball video	40	3.85 (1.75)	5.63 (1.48)
2. Hockey lose video	47	4.13 (1.66)	5.68 (1.34)
3. Hockey tie video	51	5.10 (1.51)	5.24 (1.58)
4. Hockey win video	46	4.80 (1.63)	5.00 (1.76)

### Main analysis

**Figure 1:**
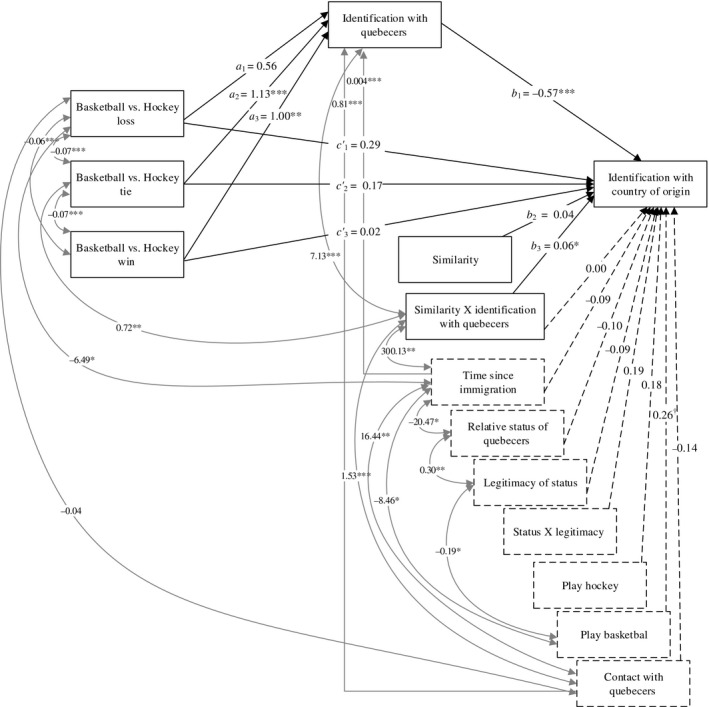
The path analysis testing the mediated moderation. *Note*. Only the significant covariances are added in the figure for the sake of simplicity. ^†^ *p* < .10; *p < .05; **p < .01; ***p < .001.

Path analysis in Mplus (with an MLR estimate) was employed to test the moderated mediation. Figure 1 illustrates the tested model. The fit indices indicate that the model fit well the data: χ^2^ (4, *N *=* *184) = 2.35 (*p *=* *.670), RMSEA = .00 (*p *=* *.820), and CFI = 1.00. As can be seen in Figure 1, our hypothesis of moderated mediation is supported by the data. Specifically, participants in the hockey loss condition did not identify more with Quebecers compared to those in the basketball condition (*a*_1_ = 0.33, *p *=* *.337). In contrast, compared to individuals who watched basketball, participants in the hockey tie condition (*a*_2_ = 1.13, *p *<* *.001) and the hockey win condition (*a*_3_ = 0.99, *p *=* *.002) identified more with Quebecers. In turn, identification with Quebecers predicted lower identification with the country of origin (*b*_1_ = −0.57, *p *<* *.001), a relation that was moderated by similarity (*b*_3_ = 0.06, *p *=* *.013).

The results also revealed the indirect effect of basketball/hockey tie and Basketball/Hockey win on identification with the country of origin via identification with Quebecers to be moderated by similarity (Basketball/Hockey tie index of moderated mediation = 0.07, *p *=* *.022; Basketball/Hockey win index of moderated mediation = 0.06, *p *=* *.035). In other words, compared to individuals who watched basketball, individuals who observed the hockey win and the hockey tie video experienced higher identification with Quebecers, which in turn predicted lower identification with their country of origin. Importantly, however, these mediations depended on the perceived level of similarity. The indirect effect of Basketball/Hockey tie on identification with country of origin was negative when similarity levels were very low (a value of 1: indirect effect = −0.58, *p *=* *.001) or medium (a value of 4: indirect effect = −0.38, *p *=* *.002), but was not different from zero when similarity levels were very high (a value of 7: indirect effect = −0.19, *p *=* *.145). A similar pattern emerged for the indirect effects of basketball/Hockey win (a value of 1: indirect effect = −0.51, *p *=* *.003; a value of 4: indirect effect = −0.34, *p *=* *.005; a value of 7: indirect effect = −0.17, *p *=* *.148). As for the indirect effect of Basketball/Hockey lose on identification with the country of origin, it was found to be not significantly different from zero (index of moderated mediation = 0.03, *p *=* *.139).2To ensure that the results held regardless of participants’ citizenship status, the same analyses were conducted but controlling for Canadian citizenship. The results were very similar to those presented in the results section. These analyses were also conducted while categorizing similarity (low, medium, and high similarity) and the same pattern of results emerged (see ‘Results with categorized similarity variable’ in Appendix S1).

### Additional analyses

To further validate the hypothesis, an opposite model was tested, where the dummy variables predict identification with the country of origin, which in turn predict identification with the country of origin in interaction with similarity. The fit of this model was slightly lower than the original model yet fit indices remained acceptable, χ^2^ (3, *N *=* *184) = 1.089 (*p *=* *.395), RMSEA = .00 (*p *=* *.494), and CFI = 1.00. As for the links in the model, two of the dummy variables (Basketball/Hockey win; Basketball/Hockey tie) and the new mediator (identification with country of origin) significantly predicted identification with Quebecers (the new dependent variable); however, the dummy variables did not predict the new mediator. As such, there was no sign of an indirect effect (all *p*s* *>* *.071) or of a moderated mediation (all indexes of moderated mediation *p*s > .237).

An additional set of post‐hoc analysis were conducted to validate whether the observed effects of watching hockey as actual participation can be distinguished from identity salience. Identity salience involves the activation of stereotypes and other social constructs that temporarily affect the cognitions and behaviours of people (e.g., DeMarree, Wheeler, & Petty, 2005; Smeesters, Wheleer & Kay, 2010) and in turn increase identification. Identities can be made salient by answering questions relevant to the group (e.g., identification scales; Hugenberg & Bodenhausen, 2004; demographic questions, McGlone & Aronson, 2006; the language of the questionnaire Lechuga, 2008; Ross, Xun, & Wilson, 2002) and by exposition to visual images (Benet‐Martinez & Haritatos, 2005). Given this, watching hockey videos (in three conditions out of four) may be conceptualized as identity salience (as opposed to participation), causing higher Quebecer identification.

To test whether watching hockey could simply be a form of identity salience, we compared the effect of the hockey and basketball conditions to the effect of another form of identity salience that took place, namely answering hockey‐related questions before the videos were presented (see Procedure section). If watching hockey simply results in a salience effect, then the effect of the conditions on Quebec identity should be similar to the effect of the hockey questions that make Quebec identity salient, resulting in two additive (or independent) effects. On the other hand, if different processes are in play when watching hockey, the effect of making the Quebec identity salient via the hockey questions should be different from watching hockey, yielding an interaction effect between the conditions and the identity salience questions. In such an instance, the salience argument for the hockey videos would be unsupported.

To test this, a simple interaction model was tested in PROCESS (Model 1; Hayes, 2013), in which identification with Quebecers was predicted by the conditions (same dummy variables as in the main analysis, with basketball as the comparison group) and the identity salience questions (as the moderator). The results do not support the salience alternative as they reveal an interaction effect; the hockey questions become non‐relevant in predicting identification with Quebecers when participants watch hockey (in the three hockey conditions). For details on the results, see ‘Results for identity salience’ in the Appendix S1. These results support an interpretation of watching hockey as different from identity salience.

## Discussion

Immigration and the identity shift it brings about are extremely complex processes. Given this reality, most research done with immigrants, particularly research in acculturation, is plagued with questions of how to successfully operationalize and measure the changes caused by immigration (Arends‐Tóth & van de Vijver, 2006; Ryder, Alden, & Paulhus, 2000; Ryder & Dere, 2006). Based on previous studies (Cárdenas & de la Sablonnière, 2017; Cárdenas *et al*., 2018), the present research tests whether engaging in behaviours typical of the host country (i.e., participating in it) can be an important source of change in immigrants’ identities (see also Klein, Spears, & Reicher, 2007). This proposition was tested by experimentally manipulating participation in the new group via watching hockey (versus watching basketball). The results offer initial support for the causal impact of participation on identity shifts.

Beyond replicating previous findings with an experimental methodology, the present research specified for the first time the conditions under which participating in a new group will impact one's identities. In line with social identity theory, it was found that individuals would not identify with a group that would add negative value to their social identity (Quebecers that lose when playing hockey). Under conditions in which the new group had a positive or a neutral value, then the general principle by which participating in the new group increased identification was indeed found. These results are also in line with a different line of research in social psychology: the phenomenon of basking in reflected glory (Cialdini *et al*., 1976). Cialdini and colleagues observed that university students self‐identified as members of the team (e.g., using the pronoun ‘we’) under conditions of victory, but disidentified (e.g., using the pronoun ‘they’) under conditions of loss. Similar findings have been obtained in the realm of political parties (Miller, 2009; Poorthuis, Thomaes, Denissen, van Aken, & Orobio de Castro, 2012), as the signs of the winning political parties are displayed in houses for longer than signs of losing parties. The results of this study offer support to this effect for cultural identities, highlighting the impact of participation in positively regarded groups on individual's cultural identities.

### Future studies

The current research is one of the few (or any, to our current knowledge) studies to experimentally study the way in which immigrants change (or acculturate) in the new cultural group. As with any experimental design, the current experiment presents a simplified version of phenomenon occurring in the real world, participating in a new group involves much more than watching a 5‐min sport video. While cognizant that the current experimental manipulation does not represent the full extent of participation in a new group, it does answer the call for a better understanding of the process by which immigrants change (Ryder & Dere, 2006). Further experimental studies manipulating other forms of participation (e.g., language, new food, and friendships) as well as longitudinal studies can offer further support for the causal effect of participation on identification with the new group and identification with the group of origin. In addition, the current study employed single‐item measures of identification (Postmes *et al*., 2013). Even though this is a reliable and valid measure of general social identification, using identification measures that capture multiple facets of this concept would allow for a closer examination of how different aspects of identification change following participation.

Further, the current sample was, on average, composed of immigrants that have lived in Canada for over 10 years. Thus, many had obtained the Canadian citizenship and had had multiple opportunities to participate in the new cultural group in the past; developing some form of identification with it before our experimental manipulation was presented. A more stringent test of our hypotheses would have been to exclusively target immigrants who have recently immigrated to Quebec. An experiment with this restricted sample would allow us to test the extent to which participation is crucial in the initial difficult years of immigration.

From a more practical perspective, government programmes designed to help immigrants adapt to the new country can include typical behaviours that are well‐valued to encourage identification with the new group. Similarly, field workers could potentially make use of these findings to encourage immigrants’ satisfaction and sense of belonging in the new group. However, if these policies are to be multicultural – as opposed to assimilationist – it is important to balance the presentation of the new cultural group with celebrating the culture of origin of immigrants (Berry & Sam, 2014). Indeed, immigrants prefer multicultural policies and practices, those that allow them to maintain their culture of origin and adopt the new culture (e.g., Lambert & Taylor, 1988). Further, multiculturalism and its practices are generally associated with greater well‐being among migrants (Arends‐Tóth & van de Vijver, 2003). Encouraging participation in the new cultural group and the group of origin could result in gaining and maintaining both cultural identities and greater well‐being in this population.

However, the presentation and celebration of both cultural groups needs to be done with care. On the one hand, our findings suggest that emphasizing differences between cultures and their practices might result in the subtractive identification pattern. On the other hand, emphasizing similarities between cultures can violate the need for distinctiveness (Hornsey & Hogg, 2000), which could have negative consequences for how identities interact with each other, resulting in discarding one or the other. A possible solution to this issue would be omniculturalism, or recognizing first and foremost the common humanity between cultural groups (Moghaddam, 2012). Recognizing the critical common similarities between groups that need to be respected may allow both cultural groups to be recognized as dissimilar yet important, remaining strong within individuals.

In conclusion, immigration is a global phenomenon that impacts the identities of millions of individuals. Studying the factors that promote identity changes allows researchers to understand the psychological mechanisms by which individuals adapt to new groups and the malleability of cultural identities.

## Supporting information


**Appendix S1**. Results with categorized similarity variable and Results for identity salience.
