# Supplementary material for: Participating in a new group and the identification processes: The quest for a positive social identity
Source: Br J Soc Psychol. 2019 Oct 11;59(1):189–208. doi: 10.1111/bjso.12340 (PMC6972616; doi:10.1111/bjso.12340)

**Supporting information**

**1. Results with categorized similarity variable**

**2.** **Results for identity salience**

**3. Table 1 of Supporting information**

**4. Figure 1 of Supporting information**

**1. Results with categorized similarity variable**

A second way in which we ensured that the non-normality (i.e., the U-shaped distribution) of similarity did not impact our results was by dividing the continuous similarity variable into a categorical variable, low similarity (scores between 1 and 3) and high similarity (scores between 5 and 7) and examining if the results were replicated with this transformed variable. Individuals who scored 4 in similarity were removed for the sake of this supplementary analysis (*n* = 18). The results with the categorical similarity were very similar to those with the continuous similarity: χ2 (4, *N* = 166) = 2.161 (*p* = .705), RMSEA = .00 (*p* = .833) and CFI = .1. Compared to individuals who watched basketball, participants watching the Canadians lose did not identify more with Quebecers (*a*_1_ =0.28, *p* = .455). However, compared to watching basketball, participants in the hockey tie condition (*a*_2_ =1.00, *p* = .002) and the hockey win condition (*a*_3_= 0.84, *p* = .015) identified more with Quebecers. In turn, identification with Quebecers predicted lower identification with the country of origin (*b*_1_ = -0.90, *p* < .001), as did the interaction between similarity and identification (*b*_3_ = 0.38, *p* = .004). Similarity did not predict identification with country of origin (*b*_2_ = 0.13, *p* = .572). Similarly to the original results, the indirect effect of Basketball/Hockey tie and Basketball/Hockey win on identification with the country of origin via identification with Quebecers was moderated by similarity (Basketball/Hockey tie index of moderated mediation = 0.38, with a 95% confidence interval ranging from 0.090 to 0.770 based on 5000 bias-corrected bootstraps; Basketball/Hockey win index of moderated mediation = 0.32, with a 95% confidence interval ranging from 0.042 to 0.696 based on 5000 bias-corrected bootstraps). Overall, the results with the categorical variable of similarity replicate those presented in the results section of the article.

**2. Results for identity salience examination**

To validate whether the observed effects of watching hockey in three out of four conditions could best be conceptualized as a form of identity salience, as opposed to actual participation, the conditions were compared to the responses to the hockey questions that make identity salience. Participants in the four conditions answered hockey related questions before the videos were presented (i.e., how often participants watched hockey, watched hockey with other Quebecers, played hockey, and whether they were fans of hockey; see Procedure section). Similar questionnaires have been previously used to make identity salient (identification scales; Hugenberg & Bodenhausen, 2004; demographic questions, McGlone & Aronson, 2006; the language of the questionnaire, Lechuga, 2008; Ross, Xun, & Wilson, 2002). In such questionnaire-based identity salience manipulations, positive answers to the questions facilitate their appropriate interpretations (i.e., assimilation effect; Bless & Burger, 2016; Smeesters, Wheleer & Kay, 2010). Identification with a group increases as a result of having an identity made salient thought positive answers to questionnaires.

If the Quebecer identity was made salient in the four conditions via de hockey questions, and if this was further made salient by watching the hockey videos (in three conditions), then we would expect positive answers to the identity salience questions to predict greater identification with Quebecers in all conditions, with participants having watched hockey identifying on average more with Quebecers than those who watched basketball (higher intercept). In other words, one would expect identity salience to have the same positive effect on identification, regardless of the medium by which it is delivered (videos or questionnaire). Thus, both effects of identity salience would add (and not interact) with each other. However, if results were to show that agreement to the identity salience questions predicts identification differently as a function of conditions (i.e., a significant interaction between conditions and the hockey questions), then the argument that watching hockey is another form of identity salience could not explain why there are different patterns of identity salience across conditions. In such instance, another interpretation of watching the hockey videos, such as conceptualizing as a form of participation, would better explain the observed results.

To test this, a simple interaction model was tested in PROCESS (Model 1; Hayes, 2013), in which identification with Quebecers was predicted by the conditions (same dummy variables as in the main analysis, with basketball as the comparison group) and the identity salience questions (as the moderator). The results of the regression model can be observed in Table 1 and Figure 1 of Supporting information (see below). There was a main effect of the hypothesized conditions (basketball versus hockey neutral; basketball versus hockey win), replicating findings from the main analyses, as well as a main effect of agreement to the identity salience questions (these were standardized and averaged for ease of interpretation). Importantly, and in line with conceptualizing watching hockey as a form of participation, the conditions and identity salience interacted together in predicting identification with Quebecers. Further, simple slope analysis reveal that only in the basketball condition did agreement with the identity salience questions predict greater identification with Quebecers (simple slope basketball condition = 1.38, *SE* = 0.39, *p* < .001; simple slope hockey loss condition = 0.38, *SE* = 0.34, *p* = .271; simple slope hockey neutral condition = 0.40, *SE* = 0.32, *p* = .207; simple slope hockey win condition = 0.00, *SE* = 0.26, *p* = .999). Thus, we do not find evidence of an additive effect of having had the Quebec identity made salient twice (via the questionnaire and the video watching conditions). Instead, these results reveal the unique effect of watching hockey, as evidenced by different slopes in each condition. This unique effect cannot be attributed to identity salience (which was present in all conditions but only had an effect in the basketball condition) nor to a floor effect (identification scale had a Likert-scale from 1 to 7). Thus, we attribute it to participation in Quebec culture that directly feeds identification with Quebecers.

**3. Table 1 of Supporting information**

| Table 1. Moderation analysis of identity salience and condition on identification with Quebecers | | | | | | |
| --- | --- | --- | --- | --- | --- | --- |
|  |  | Identification with Quebecers (Y) | | | | |
| Independent variables |  | Unstandarized beta |  | *SE* |  | *p* |
| 1. Condition (Basketball is comparison group) |  |  |  |  |  |  |
| Hockey loss (X1) |  | 0.33 |  | 0.34 |  | .337 |
| Hockey neutral (X2) |  | 0.98 |  | 0.34 |  | .005 |
| Hockey win (X3) |  | 1.26 |  | 0.34 |  | <.001 |
| 2. Identity salience (standardized; W) |  | 1.39 |  | 0.40 |  | <.001 |
| 3.Interactions |  |  |  |  |  |  |
| Hockey loss * identity salience  (X1*W) |  | -1.01 |  | 0.52 |  | .055 |
| Hockey neutral * identity salience  (X2*W) |  | -0.99 |  | 0.51 |  | .054 |
| Hockey win * identity salience  (X3*W) |  | -1.38 |  | 0.47 |  | .004 |
| Total R^2^ = .16***; R^2^ of interactions = .04* | | | | | | |
| Notes. * p < .05, ** p < .01, *** p < .001. | | | | | | |

**4. Figure 1 of Supporting information**

Figure 1. The effect of identity salience on identification with Quebecers per condition.


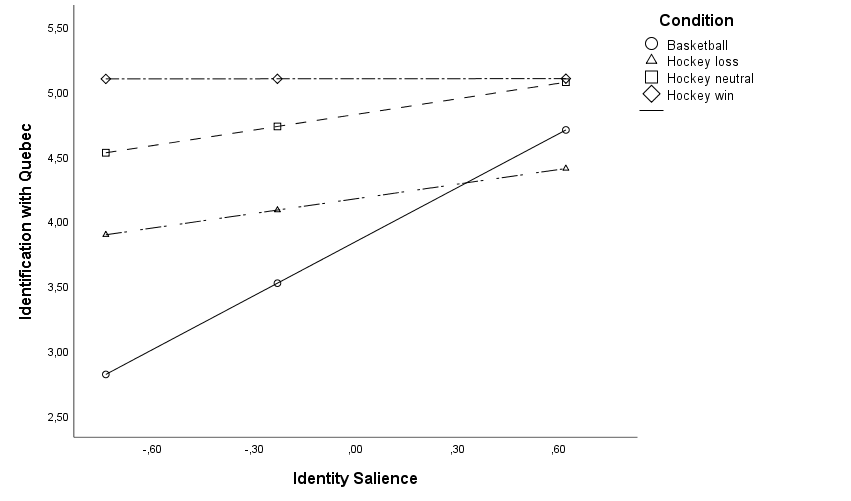

Supplement: Supplementary file 1 — Appendix S1. Results with categorized similarity variable and Results for identity salience. [file BJSO-59-189-s001.docx]
